# Dynamics of Cytokines and Chemokines During the Peripartum Period in People Living With Human Immunodeficiency Virus

**DOI:** 10.1111/aji.70147

**Published:** 2025-08-22

**Authors:** Jacqueline Corry, Natalia Zotova, Martine Tabala, Christina K. Cotrone, Fidéle Lumande Kasindi, Bienvenu Lebwaze Massamba, Pelagie Babakazo, Namal P. M. Liyanage, Nicholas T. Funderburg, Marcel Yotebieng, Jesse J. Kwiek

**Affiliations:** ^1^ Department of Microbiology The Ohio State University Columbus Ohio USA; ^2^ Infectious Diseases Institute The Ohio State University Columbus Ohio USA; ^3^ Division of General Internal Medicine Department of Medicine Albert Einstein College of Medicine Bronx New York USA; ^4^ School of Public Health University of Kinshasa Kinshasa The Democratic Republic of the Congo; ^5^ Mission and Outreach Team Hyde Park Community United Methodist Church Cincinnati Ohio USA; ^6^ Department of Pathology University of Kinshasa Kinshasa The Democratic Republic of the Congo; ^7^ Center for Retrovirus Research The Ohio State University Columbus Ohio USA; ^8^ Department of Microbial Infection and Immunity College of Medicine Ohio State University Columbus Ohio USA; ^9^ Department of Veterinary Biosciences College of Veterinary Medicine Ohio State University Columbus Ohio USA; ^10^ Division of Medical Laboratory Science School of Health and Rehabilitation Sciences The Ohio State University Columbus Ohio USA

**Keywords:** chemokine, cytokine, HIV, immune factor, peripartum, postpartum, pregnancy

## Abstract

**Problem:**

Pregnancy requires a precisely regulated immune response to support fetal development and minimize complications. Human immunodeficiency virus (HIV) infection induces a chronic inflammatory state and is associated with adverse pregnancy outcomes. In this observational cohort of pregnant people living with HIV in the Democratic Republic of the Congo (DRC), we sought to gain a deeper understanding of peripartum changes in cytokines, chemokines and soluble factors (collectively termed immune factors).

**Method of Study:**

Pregnant individuals living with HIV were enrolled during their second or third trimester in Kinshasa, DRC, between October 2020 and May 2021. Peripheral blood samples were collected at: enrollment (second or third trimester), 1–3 days postdelivery and postpartum. Concentrations of 45 immune factors were measured using LegendPlex and ELISAs.

**Results:**

Most chemokines decreased significantly from enrollment to postdelivery, followed by a rebound in the postpartum period. Meanwhile, concentrations of myoglobin, serum amyloid A‐1 protein (SAA), and interleukin 6 (IL‐6), increased from enrollment to postdelivery, followed by a decrease postpartum. Finally, protein S100‐A8/protein S100‐A9 (S100A8/A9) and insulin‐like growth factor‐binding protein 4 (IGFBP4) consistently increased from enrollment through postpartum.

**Conclusions:**

The postdelivery decline in chemokines observed in this study has not previously been reported. This shift may result from two mechanisms: greater‐than‐expected placental chemokine production or the degradation of key signaling molecules during parturition and early uterine involution. Additionally, the rise in proinflammatory markers from enrollment to postdelivery suggests a persistent inflammatory state, either unaffected by fetal delivery or worsened by tissue damage in the gestational parent.

## 1 Introduction

In 2021, preterm birth complications and HIV/AIDS ranked among the top 10 leading causes of death in low‐income countries [1]. By the end of 2023, an estimated 39.9 million people were living with human immunodeficiency virus (HIV), with women and girls comprising more than half of this population [2, 3]. In sub‐Saharan Africa over 60% of new HIV infections occur in females, with females of child bearing age being most affected [2, 4]. Pregnant individuals living with HIV are at heightened risks of adverse outcomes as compared to those living without HIV, including vertical HIV transmission, preterm birth, low birthweight, small‐for‐gestational‐age, stillbirth, and maternal, neonatal or infant mortality [5, 6, 7, 8, 9, 10, 11, 12, 13, 14, 15, 16, 17, 18, 19, 20, 21]. While public health interventions and antiretroviral therapy (ART) have been effective in reducing vertical transmission of HIV, challenges persist in preventing adverse outcomes such as low birth weight and preterm birth [8, 9, 10, 11, 12, 21, 22, 23, 24].

Pregnancy and HIV both affect the immune system, but in distinct ways. Initial HIV infection leads to a primary viremia and mild symptoms that may be similar to a variety of viral diseases [25]. Untreated or treatment refractory HIV eventually depletes CD4+ T‐cells to a point at which the individual is more susceptible to opportunistic infections and death [25, 26]. ART decreases the viral load and increases the T‐cell counts [27], but it is clear that even in treated individuals, HIV leads to gut barrier dysfunction, intestinal microbial translocation, and chronic inflammation [26, 27, 28, 29, 30]. This inflammation in multiple immune compartments is driven both by innate and adaptive immune cells [27].

Meanwhile, pregnancy requires precise immune regulation, as the fetus is an allograft. The immune system of both the gestational parent and the fetus are finely tuned to provide a particular response at a right time and place [31, 32, 33]. Gestational parental cytokines and chemokines play critical roles in all stages of pregnancy, influencing immune cell movement and phenotypic changes [31, 32, 33]. In healthy pregnancies, circulating cytokines, chemokines, and soluble factors (collectively: immune factors) change over the course of pregnancy [34]; however, complications can disrupt this balance [35, 36, 37]. Potentially as a consequence of these immune alterations, pregnant individuals have an increased susceptibility to some pathogens, while infections with other pathogens can become more severe when acquired during pregnancy [33].

Proper immune regulation is therefore critical in mitigating adverse birth outcomes and ensuring maternal and fetal well‐being [38, 39, 40, 41]. In addition, immune modulation in the pregnant individual continues to change postdelivery into the postpartum period [42, 43, 44, 45, 46, 47, 48], potentially contributing to poor gestational parental outcomes [43, 44, 45]. Despite these critical immune dynamics, research on soluble immunity during the late peripartum period in individuals living with HIV on ART remains limited [49, 50, 51, 52, 53, 54, 55]. Therefore, this study aims to characterize the soluble immune profiles of pregnant individuals living with HIV during and just after pregnancy.

## 2 Methods

### 2.1 Study Setting

This study was an observational cohort study nested within the larger randomized control trial for the Continuous Quality Improvement‐Prevention of Mother to Child Transmission (CQI‐PMCT) [56, 57, 58, 59, 60, 61, 62]. The parent study was an open label, parallel, group randomized trial designed to evaluate continuous quality improvements (CQI) on long‐term anti‐retroviral therapies (ART) outcomes among pregnant and chestfeeding individuals receiving care in Kinshasa province.

### 2.2 Study Design and Participants

All pregnant individuals, in the second or third trimester and living with HIV and receiving care at any of the selected maternal and child health facilities between October 2020 and May 2021, were eligible for the study. After obtaining written consent, 199 participants were enrolled, 81 were excluded from the final analysis for the following reasons: only one plasma sample was available for analysis (*n* = 28), missing detailed information about the delivery, which included a combination of birth weight, baby's sex, mode of delivery and information on the placenta and umbilical cord (*n* = 48), and multiple birth (*n* = 5). The final number of participants included in this analysis was 118.

Peripheral venous blood was drawn, and questionnaires were administered at enrollment, 1–3 days following delivery and approximately 6 weeks postpartum by trained personnel. Blood was drawn in EDTA containing tubes. These were centrifuged for 10 min at 200 × *g*, and plasma was aliquoted and stored at –80°C. Blood was also spotted onto Whatman paper and subjected to quantitative PCR for HIV viral load as previously described [62].

### 2.3 Definitions

Cytokines are peptides or protein secreted by cells that can have autocrine, paracrine, or endocrine effects on cells with the requisite receptor(s). Chemokines are a type of cytokine that induce directional movement along a gradient. Soluble factors are those peptides or proteins that either do not have a receptor or are not cytokines. In this manuscript, immune factor is the collective name for cytokines, chemokines, and soluble factors. Last menstrual period was self‐reported at enrollment. Gestational age was based on either last menstrual period (*n* = 95), if available, or clinical estimate, if not (*n* = 23). Enrollment (E) occurred between gestational week 15 and 40. The postdelivery (PD) blood draw occurred between 1 and 3 days postdelivery. The postpartum (PP) blood draw occurred 1–13 weeks postdelivery, with a median of 6.7 weeks and a 95% confidence interval of 6–10 weeks. The technical limit of detection for HIV RNA was <40 HIV RNA copies/mL of blood, (see below for methods). Viral suppression was defined as <1000 copies/mL of blood [63]. Eutocic delivery was one that was spontaneous and required no intervention. Alternatively, a dystocic delivery was any delivery where difficulties arose, particularly with uterine contractions, cervical dilation, the descent and engagement of the baby in the pelvis, and the baby's position during delivery, thus requiring medical or surgical intervention for the mother to deliver safely but did not require an emergent Cesarean section. All Cesarean sections were emergent. A birth was considered preterm if the neonate (newborn up to 28 days old) was born alive before 37 weeks gestational age. Death in utero was considered the death of a fetus at any point during pregnancy prior to birth, while stillbirth is death of the fetus only after gestational week 28 but before or during birth [64]. Low birth weight neonates are those that are born ≤2500 g [65].

### 2.4 Specimen Processing and Laboratory Testing

Once all plasma samples were collected, they were shipped to the United States on dry ice and stored in –80°C at The Ohio State University in Columbus, Ohio. Prior to assaying, participant's plasma samples were thawed, centrifuged at 2500 × *g* for 5 min twice to remove particulates, aliquoted, and stored at –80°C.

All immune factor names, abbreviations, and measurement units (ng/mL or pg/mL) can be found in Table S1. Immune factor names and abbreviations were curated from uniport.org, *homo sapiens*, following the International Protein Nomenclature Guidelines [66]. Thirteen chemokines were simultaneously measured in plasma using LEGENDplex Human Proinflammatory Chemokine Panel 1 (BioLegend, 740984) (interleukin‐8 (IL‐8), C‐X‐C motif chemokine 10 (CXCL10), eotaxin (CCL11), C‐C motif chemokine 17 (CCL17), CCL2, CCL5, CCL3, CXCL9, CXCL5, CCL20, CXCL1, CXCL11, CCL4) according to the manufacturer's filter plate protocol. Similarly, 12 cytokines were measured in plasma using the LEGENDplex COVID‐19 Cytokine Storm Panel 1 & 2 (BioLegend, 741095) (IL‐6, interferon alpha‐2 (IFN‐α‐2), IL‐2, IFN‐ɣ, IL‐1RN, tumor necrosis factor (TNF‐ α), IL‐10, granulocyte‐macrophage colony‐stimulating factor (GM‐CSF), IL‐1β, vascular endothelial growth factor a, long form (L‐VEGF), IL‐18, IL‐15). Thirteen cytokines and soluble factors were measured in plasma using the LEGENDplex Vascular Inflammation panel 1 (BioLegend, 740551) (myoglobin (MB), protein S100‐A8/protein S100‐A9 (S100A8/A9), neutrophil gelatinase‐associated lipocalin (NGAL), c‐reactive protein (CRP), 72 kDa type IV collagenase (MMP‐2), osteopontin (SPP1), myeloperoxidase (MPO), serum amyloid A‐1 protein (SAA), insulin‐like growth factor‐binding protein 4 (IGFBP4), soluble intercellular adhesion molecule 1 (sICAM1), soluble vascular cell adhesion protein 1 (sVCAM1), matrix metalloproteinase‐9 (MMP‐9), cystatin‐C (CST3)). Data were collected on the MACS Quant 10 Flow cytometer (Miltenyi Biotech) and using MacsQuant Software (Miltenyi Biotech). Gates were set around beads to exclude debris, and number of events collected was 300 per analyte multiplied by 1.1. Gates around individual bead sets and the corresponding gates around individual analytes were adjusted post‐collection in the LEGENDplex online Qognit software based on standards; gates were then applied to all standards and samples for a particular plate and five‐parameter logistic standard curves were generated for all. Concentrations were interpolated from these curves using the LEGENDplex Qognit software (BioLegend, Qognit Inc.).

Seven immune factors were individually measured by duoset ELISA (R&D Systems): CXCL13 (DY801), IL‐4 (DY204), IFN‐λ‐1(DY7246), soluble monocyte differentiation antigen CD14 (sCD14, DY383), soluble scavenger receptor cysteine‐rich type 1 protein M130 (sCD163, DY1607), soluble tumor necrosis factor receptor superfamily member 1A (sTNFRSF1A, DY225), sTNFRSF1B (DY726) according to the manufacturer's instructions with minor modifications. First half‐well plates were used (Greiner, 675061), plates were washed using the CAPP wash 12 (Pipette.com, W‐12) attached to a carboy that was washed daily, and plates were developed using TMB substrate Plus liquid (VWR, 97063–666). Absorbance were read on a SpectraMax i3x (Molecular Devices).

For all immune factor assays performed there were three samples that were on all plates to serve as quality control to ensure similar assay performance across plates and days. See Table S2 for dilution factors and upper and lower limits of detection (LoD). Values below the limit of detection or below the bottom standard were increased to the bottom standard, unless the dilution factor was >2, then the bottom standard was multiplied by the dilution factor. Values above the limit of detection were replaced by the top standard multiplied by the dilution factor multiplied by 1.1 (Tables S1 and S2).

### 2.5 Statistical Analyses

All graphs were constructed, and statistical tests were performed in Prism versions 9.5.1 to 10.4.1 on both Windows and Mac OS (GraphPad). Significance was tested using the following tests, as stated in the figure legends: Kruskal–Wallis test with a Dunn's correction for multiple comparisons, Mann–Whitney test, Kruskal–Wallis uncorrected for multiple comparisons, and Spearman Correlation. As this is an exploratory study, we did not correct for multiple hypothesis testing for all findings [67]. Spearman's rank correlation significance cutoffs were (*r_s_* < 0.2 or >–0.2, *p* < 0.05).

## 3 Results

### 3.1 Participant Characteristics

A total of 118 pregnant individuals living with HIV were recruited during their second or third trimester. The median age at enrollment was 33 years (IQR: 28–36 years). Among them, 79% of pregnancies resulted in eutocia, 19% in cesarean delivery, and 2% in dystocia. Overall, 110 out of 118 pregnancies resulted in live births. At delivery, only 21% of participants had detectable HIV RNA copies/mL. The majority (73%) were receiving ART consisting of tenofovir (TDF), lamivudine (3TC), and dolutegravir (DTG), while 25% were on a regimen of TDF, 3TC, and efavirenz (EFZ); treatment data was unavailable for the remaining 2%. Most (80%) participants were multigravida with a median of four previous pregnancies. Of those, 61% had previously experienced the loss of a fetus, neonate, infant, toddler, or multiple losses (Table 1).

**TABLE 1:** Obstetrical data and outcome.

Variable	*N* (%)	Median (IQR)
Participant age (years)	118 (100)	33 (28, 36)
Gestational week at enrollment		29 (24, 33)
Gestational week at delivery		39 (36, 40)
Postpartum sample collection (weeks)		6.7 (6.4, 7.3)
Gravidity		
Primigravida	24 (20)	
Multigravida	94 (80)	
Parity		
Nulliparous	33 (28)	
Primiparous	27 (23)	
Multiparous	58 (49)	
Previous pregnancies outcome^a^		
Death in utero	41 (44)	
Neonatal death	14 (15)	
Infant death	8 (9)	
Toddler death	6 (6)	
Viral load at delivery		
Detectable (≥40 HIV copies/mL)	25 (21)	
Undetectable	93 (79)	
ART		
TDF+3TC+DTG	85 (72)	
TDF+3TC+EFV	30 (25)	
Missing	3 (3)	
Infant sex		
Male	57 (48)	
Female	61 (52)	
Infant weight (g)		3080 (2820, 3400)
≤2500 g	9 (8)	
>2500 g	99 (84)	
Missing	10 (8)	
Other adverse outcomes^b^		
Preterm birth	35 (30)	
Stillbirth	8 (7)	
Neonatal death	2 (2)	

Abbreviations: 3TC, lamivudine; DTG, dolutegravir; EFV, efavirenz; TDF, tenofovir.

^a^ Percentages do not add up to 100%—categories not exclusive, some individuals with multiple pregnancies had different outcomes.

^b^ Percentages do not add up to 100%.

### 3.2 Chemokines Decrease Postdelivery and Rebound Postpartum

**FIGURE 1:**
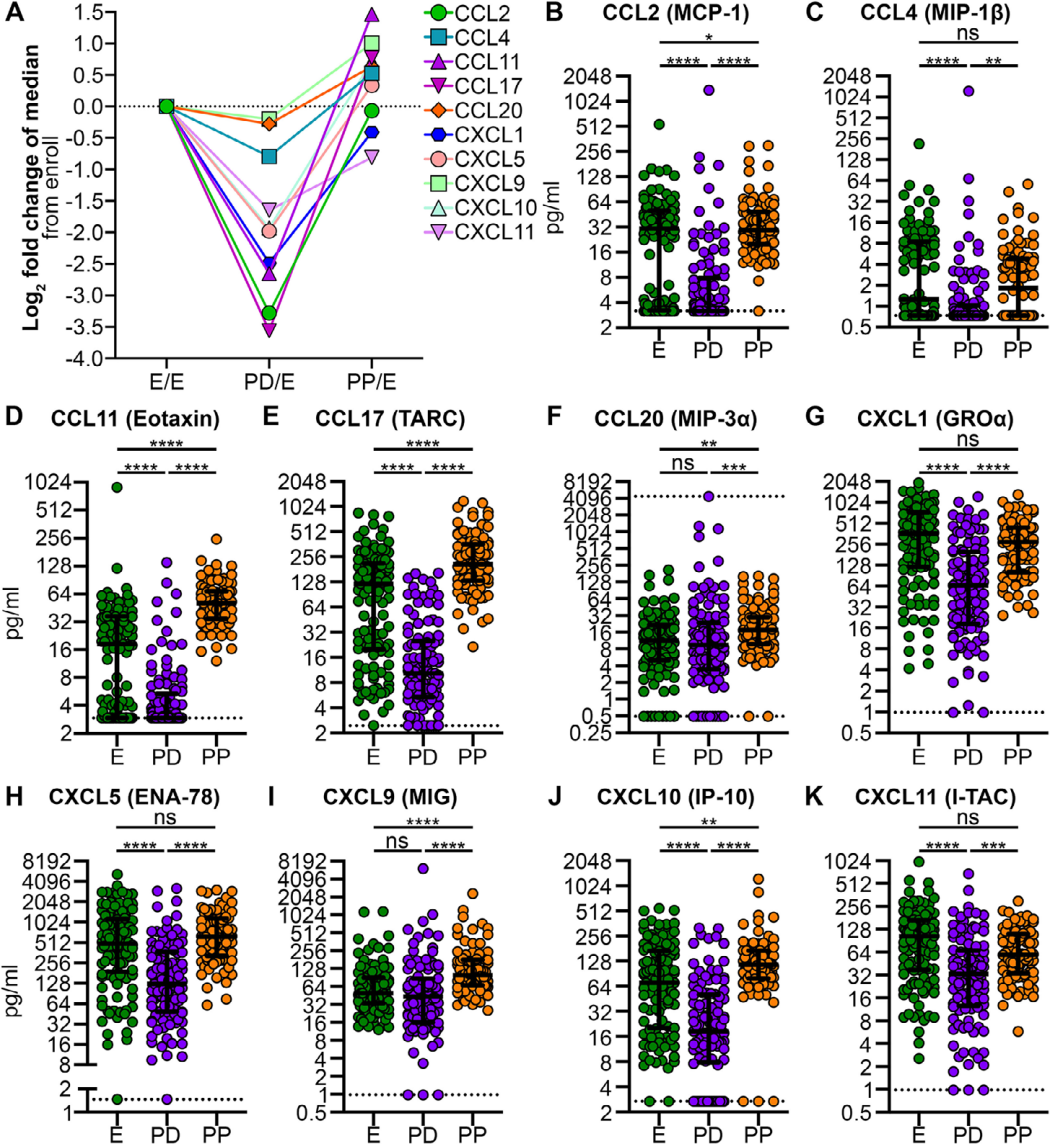
Chemokines decrease from enrollment to postdelivery and increase from postdelivery to postpartum. Chemokines were measured in plasma collected from participants at enrollment (E), postdelivery (PD), and postpartum (PP) using LEGENDplex assays. (A) Log_2_ fold change of the median values of chemokines from the enrollment time point. (B–K) Each dot represents an individual participant's analyte measurement, with horizontal lines indicating the median, upper and lower quartiles. Dotted lines represent limits of detection (LoD), see Table S2 for all LoD values. Statistical significance tested by Kruskal–Wallis with a Dunn's correction for multiple comparison. ns, not significant, *p < 0.05, **p < 0.01, ***p < 0.001, ****p < 0.0001.

**FIGURE 2:**
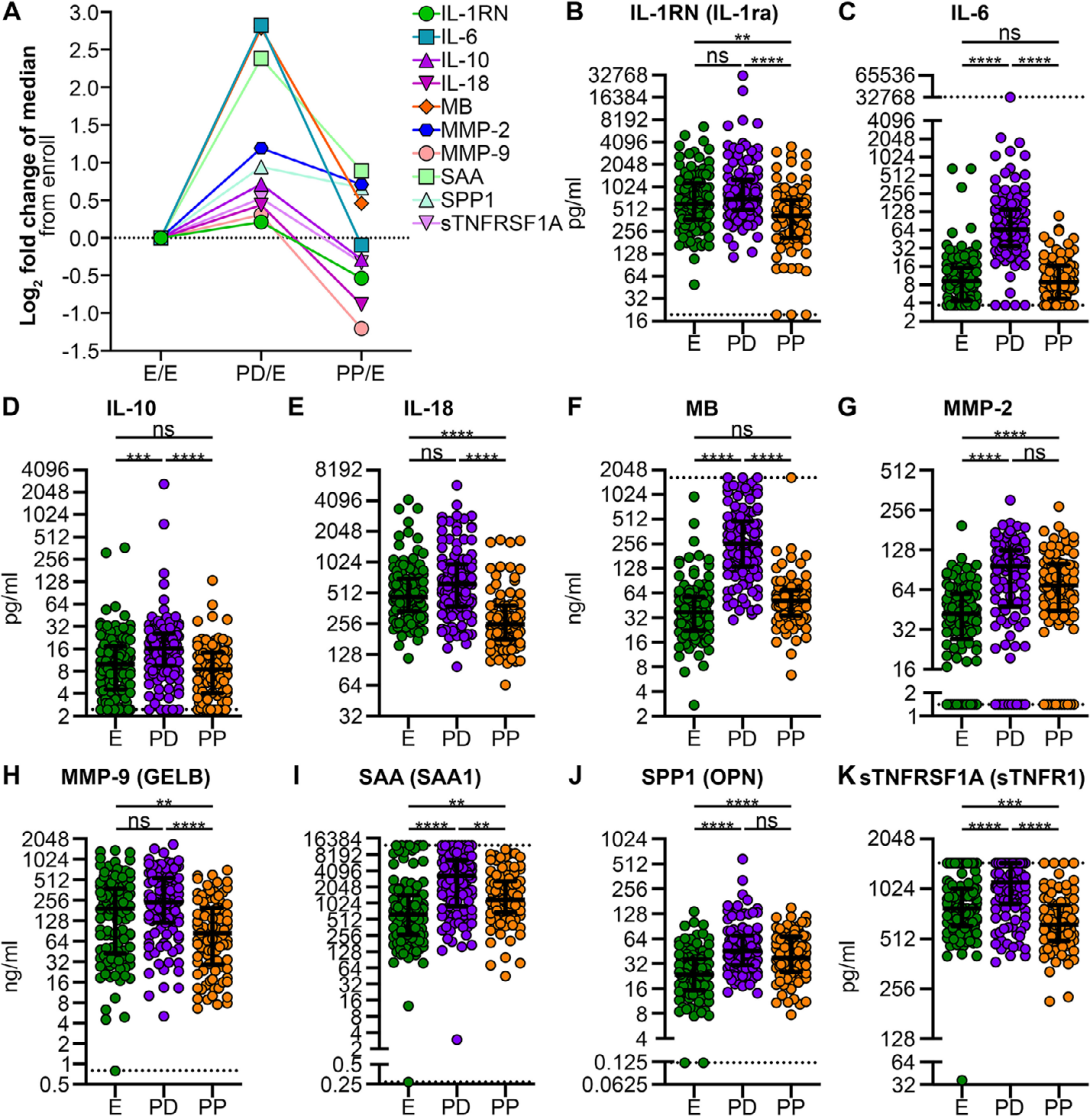
Cytokines and soluble factors that increase from enrollment to postdelivery and decrease postpartum. Cytokines and soluble factors were measured in plasma collected from participants at enrollment (E), postdelivery (PD), and postpartum (PP) using LEGENDplex assays (B–J) or ELISA (K). (A) Log_2_ fold change of the median values of all cytokines and soluble factors from the enrollment time point. Immune factor concentrations plotted as pg/mL (B–E, K) or ng/mL (F–J). (B–K) Each dot represents an individual participant's analyte measurement, with horizontal lines indicating the median, upper and lower quartiles. Dotted lines represent LoD, see Table S2 for all LoD values. Statistical significance tested by Kruskal–Wallis with a Dunn's correction for multiple comparison. ns, not significant, *p < 0.05, **p < 0.01, ***p < 0.001, ****p < 0.0001.

**FIGURE 3:**
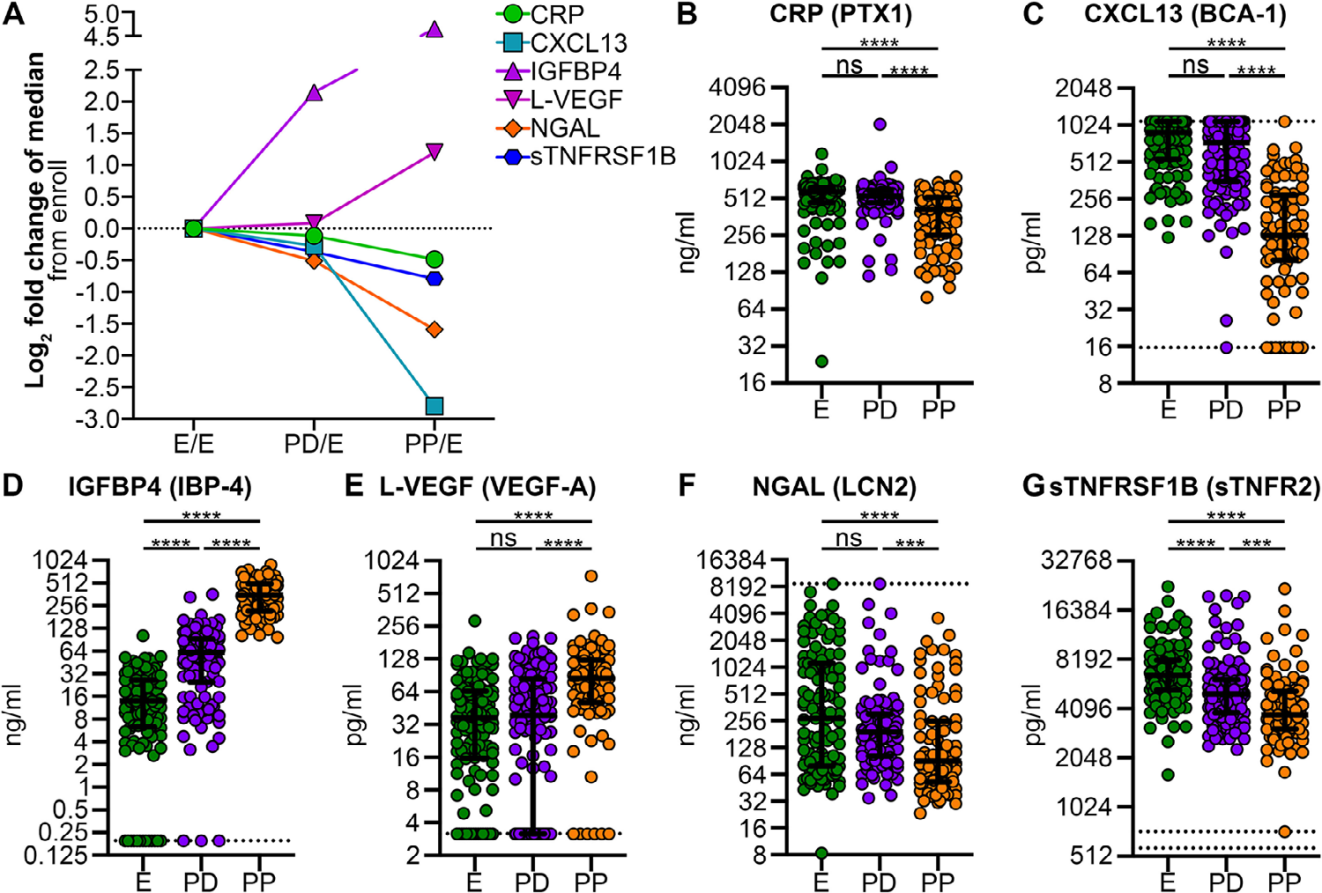
Immune factors that increase or decrease from enrollment to postdelivery and continue in the same direction postpartum. (A–G) Immune factors were measured in plasma collected from participants at enrollment (E), postdelivery (PD), and postpartum (PP) using LEGENDplex assays (B, D–F) or ELISA (C, G). (A) Log_2_ fold change of the median values of all immune factors from the enrollment time point. Immune factor concentrations plotted as pg/mL (C, E, G) or ng/mL (B, D, F). (B–G) Each dot represents an individual participant's analyte measurement, with horizontal lines indicating the median, upper and lower quartiles. Dotted lines represent LoD, see Table S2 for all LoD values. (D) Top dotted line is the lower LoD for enrollment and postpartum, bottom dotted line is LoD postdelivery. (B–G) Statistical significance tested by Kruskal–Wallis with a Dunn's correction for multiple comparison. ns, not significant, ***p < 0.001, ****p < 0.0001.

**FIGURE 4:**
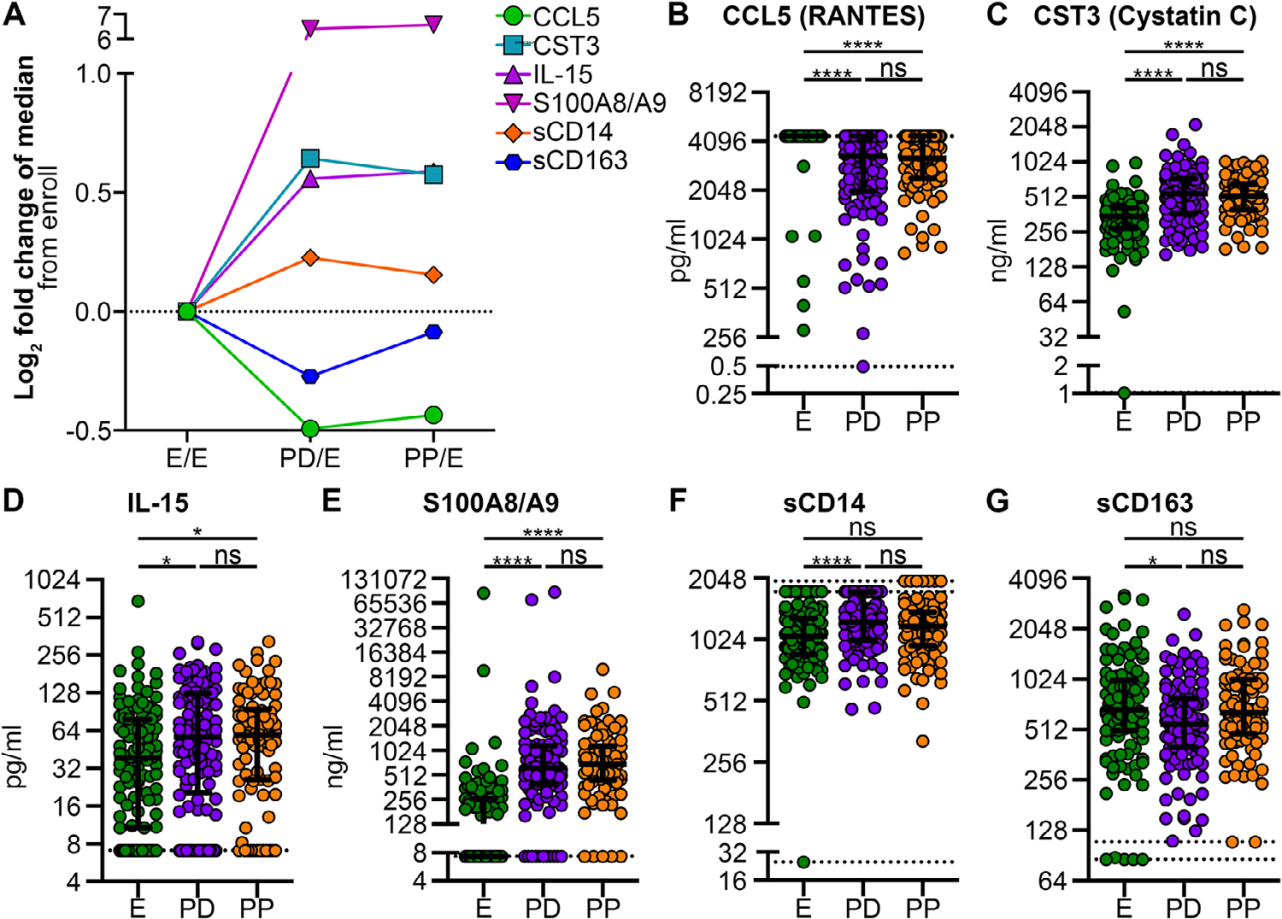
Immune factors that increase or decrease from enrollment to postdelivery and remain constant from postdelivery to postpartum. (A–G) Immune factors were measured in plasma collected from participants at enrollment (E), postdelivery (PD), and postpartum (PP) using LEGENDplex assays (B–E) or ELISA (F, G). (A) Log_2_ fold change of the median values of all chemokines and soluble factors from the enrollment time point. Immune factors plotted as pg/mL (B, D) or ng/mL (C, E–G). (B–G) Each dot represents an individual participant's analyte measurement, with horizontal lines indicating the median, upper and lower quartiles. Dotted lines represent LoD, see Table S2 for all LoD values. (F) Top dotted line is the upper LoD for postpartum, the line below is the upper LoD for enrollment and postdelivery, the bottom dotted line is LoD for enrollment. (G) Bottom dotted line is the lower LoD for enrollment and postdelivery, line above is the lower LoD for postpartum. (B–G) Statistical significance tested by Kruskal–Wallis with a Dunn's correction for multiple comparison. ns, not significant, *p < 0.05, **p < 0.01, ***p < 0.001, ****p < 0.0001.

To observe temporal changes in immune factors in individuals living with HIV during and after pregnancy, immune factors were normalized to the enrollment and grouped by how each changed relative to enrollment. While some immune factors remained relatively stable throughout pregnancy (Figure S1), several immune factors showed significant changes relative to their enrollment timepoint (Figures 1, 2, 3A, and 4A). Several chemokines decrease from enrollment to postdelivery and then rise again postpartum, with most returning to near enrollment levels. Some chemokines exhibit a more pronounced decline between enrollment and postdelivery, including CCL17 (12‐fold), CCL2 (10‐fold), CCL11 (6‐fold), and CXCL1 (6‐fold). The greatest increases from postdelivery to postpartum were observed in CCL17 (20‐fold) and CCL11 (17‐fold) (Figure 1 and Table S3).

### 3.3 Immune Factors Increase Postdelivery Then Decrease Postpartum

Several cytokines and soluble factors increase postdelivery and then decline postpartum. IL‐6 (7‐fold) and MB (7‐fold) had the largest postdelivery increases among immune factors that later returned to enrollment levels postpartum. SAA exhibited a more moderate 5‐fold postdelivery increase, followed by a two‐fold decrease postpartum. Several other cytokines and soluble factors, including IL‐10, MMP‐2, and SPP1, also showed smaller, yet statistically significant increases from enrollment to postdelivery. Although MMP‐9 showed only a minimal increase postdelivery, its levels decreased threefold from postdelivery to postpartum (Figure 2 and Table S3).

### 3.4 Immune Factors Increase or Decrease Post‐Enrollment

CXCL13 and NGAL showed no significant change from enrollment to postdelivery, but decreased 7‐fold and 3‐fold, respectively, from enrollment to postpartum. Conversely, IGFBP4 increased fourfold from enrollment to postdelivery and sixfold from postdelivery to postpartum (Figure 3 and Table S3). S100A8/A9 increased the most of all factors tested, increasing 85‐fold from enrollment to postdelivery, followed by a 1‐fold increase postpartum (Figure 4 and Table S3).

### 3.5 Immune Factors Associated With HIV and ART

To test if immune factors varied by HIV quantity in the blood, we first compared individuals with undetectable and detectable viral loads. There were very minor but significant differences across all time points, but the ones with the largest differences were all postpartum, where CCL4 (4‐fold), CCL2 (2‐fold), CXCL5 (2‐fold), and IL‐10 (2‐fold) had lower expression in individuals with detectable viral loads (Figure S2). As a sensitivity analysis, we also compared the immune factors of individuals with viral suppression or undetectable viral loads with those that were unsuppressed, and we observed that the following immune factors were associated with detectable viral load: IL‐1RN at enrollment, CCL4 postdelivery, and CXCL5 postpartum (Figure S3). When comparing participants on a DTG‐based versus an EFV‐based regimen, no significant differences were observed at enrollment. However, postdelivery and postpartum several cytokines were significantly elevated in those on a DTG‐based regimen, IFN‐γ (5‐fold PD, 2‐fold PP), L‐VEGF (3‐fold PD, 2‐fold PP), IL‐10 (2‐fold PP), and IL‐18 (2‐fold PD). IGFBP4 (2‐fold PD) is the only soluble factor that is significantly higher in those treated with an EFZ‐based regimen (Figure S4).

### 3.6 Temporal Correlation of Immune Factors

To test if the immune factors at enrollment were affected by the time to delivery or timing of enrollment, the concentrations were plotted against the time between enrollment and delivery in weeks. The immune factors that are most highly correlated with time to delivery by Spearman rank correlation are IGFBP4, MPO, sTNFRSF1A, and NGAL. All others were either weakly (Figure S5) or not significantly (Figures S6 and S7) correlated. Similar results were observed with correlations between gestational age and immune factor concentration with IGFBP4, MPO, NGAL, and MMP‐9 being the most highly correlated (Figure S8). Using Spearman's rank correlation, both positive and negative correlations were noted between the immune factors at enrollment (Figure S9 and Table S4), postdelivery (Figure S10 and Table S5), and postpartum (Figure S11 and Table S6). There were more and stronger positive correlations than negative correlations at all‐time points.

## 4 Discussion

In this secondary analysis of a larger cohort of pregnant individuals living with HIV, enrolled between October 2020 and May 2021, we quantified immune factors in plasma at three time points: enrollment, postdelivery, and postpartum. Our goal was to better understand the dynamics of these immune factors throughout pregnancy and postpartum. We observed that while most chemokines decreased significantly from enrollment to postdelivery, inflammatory cytokines and soluble factors associated with inflammation, such as IL‐6 and SAA, increased during this period. Although most immune factor concentrations returned to levels similar to those at enrollment by the postpartum period, IGFBP4 and S100A8/A9 remained elevated relative to postpartum.

At the end of pregnancy, there is an acute decrease in blood volume due to the expulsion of the placenta during both vaginal and cesarean deliveries [68, 69]. Simultaneously, significant hormonal changes occur, including increases in prolactin, oxytocin and cortisol alongside decreases in progesterone and estrogen [70, 71]. The loss of the placenta and the physical trauma of childbirth result in extensive muscular and tissue damage that requires repair. Collectively, these factors would suggest that the process of labor and delivery is an inherently inflammatory process. Consistent with this, among the most pronounced systemic increases observed between enrollment and postdelivery were the proinflammatory molecules S100A8/A9, IL‐6 and SAA, and MB, a marker of muscle damage or oxygen deficiency. Both MB and IL‐6 had previously been reported to rise during labor and delivery [42, 43, 45, 72]. However, to our knowledge, no studies have examined SAA and S100A8/A9 levels in the same individual during and after pregnancy [73, 74, 75, 76].

Among the immune factors analyzed, S100A8/A9 and IGFBP4 were the only two factors that increased postdelivery and remained elevated into the postpartum period. Notably, IGFBP4 concentrations at enrollment were significantly and inversely associated with time to delivery across participants, likely because PAPP‐A, a metalloproteinase that circulates at high levels during pregnancy, is known to cleave IGFBP4 [77, 78]. The IGFBP4 concentrations observed during pregnancy align with previous findings, while postpartum levels in our participants were consistent with control populations in other studies [79, 80, 81]. This suggests a suppression during pregnancy, and the postpartum increase reflects a likely return to normal values. Similarly, though the values for postpartum circulating S100A8/A9 are unavailable, control data indicate that levels in these participants remain elevated [82, 83, 84]. However, since pre‐pregnancy samples for these participants are not available, it is unclear how these levels compare to baseline. A recent study by Ross et al. suggests that immune factor trajectories during and after pregnancy are unique, influenced by host factors, and, in some cases, can be affected for at least a year postdelivery [85].

While IGFBP4 and S100A8/A9 remained elevated postpartum, other immune factors exhibited different trajectories. Chemokines followed a distinct pattern, with most decreasing postdelivery despite a concurrent rise in inflammatory markers. Chemokines play a crucial role in coordinating leukocyte migration, trafficking, and activation via receptor interactions [86]. To our knowledge, this phenomenon has not been previously reported, though it is uncertain whether prior studies have examined these specific time points or chemokine profiles. One possible explanation is that the placenta serves as a major source of circulating chemokines [87, 88, 89], and its removal leads to a decline as these molecules degrade or are cleared from circulation. One factor that complicates this argument is the weak but significant correlation between several chemokines and time to delivery (Figure S5). Among the chemokines analyzed, CCL2, CCL11, and CCL17 exhibited the most significant changes from enrollment to postdelivery and/or from postdelivery to postpartum. While considerable research exists on circulating CCL2 levels during pregnancy, findings are inconsistent across studies [34, 41, 73, 87, 89, 90, 91, 92, 93]. Data on CCL11 [34, 41, 90] and CCL17 [47, 94, 95] are more limited. Our findings align with previously reported CCL11 levels [34, 41, 90], while the CCL17 data are more consistent with some studies than others [47, 94, 95]. Notably our measured concentrations most closely match a study that also utilized a multiplex assay, using a different platform [34].

In addition to chemokines and their distinct peripartum dynamics, other immune factors involved in immune regulation, tissue remodeling, and placental development—namely MMP‐2 and MMP‐9—demonstrated lower values than in the published literature [96, 97]. Dysregulation of MMP‐2 and/or MMP‐9 has been implicated in both gestational hypertension and preeclampsia [98]. However, our cohort lacks data on gestational or postpartum hypertension and preeclampsia, limiting direct comparisons. Existing evidence suggests no difference in the prevalence of preeclampsia or gestational‐associated hypertension in individuals living with HIV and those without [24, 99]. Therefore, based on prior studies, we, we could potentially expect to have approximately 5%–6% of participants with hypertensive disorders of pregnancy [6, 100]. This underscores the importance of collecting gestational parental health information throughout pregnancy and postpartum, alongside monitoring fetal, neonatal and infant health outcomes.

Ours study also observed cytokines with values higher than those reported in the literature. We analyzed three cytokines from the IL‐1 family: IL‐1β, IL‐1RN, and IL‐18. At enrollment, the concentrations of all three were higher than those reported in the literature [34, 75, 90, 91, 92, 93, 101, 102]; however, while IL‐18 and IL‐1RN values are all within the limits of detection for the assay, 35% of the participants had undetectable levels of IL‐1β. Both IL‐1β and IL‐18 are produced as inactive precursors and are activated via cleavage by proteins in the inflammasome complex [103, 104]. However, they are expressed by different cells [105], which may explain why IL‐18 concentrations in our cohort were markedly elevated compared to available data [90, 92, 106], while IL‐1β levels remained relatively low [34, 75, 90, 91, 92, 93, 101, 102]. Although both IL‐1β and IL‐18 are potently pro‐inflammatory mediators [105], their regulation differs: IL‐1RN antagonizes the activities of IL‐1β, but not IL‐18 [107]. In vitro studies suggest IL‐1RN concentrations may need to exceed IL‐1β concentrations 10‐100‐fold to effectively inhibit IL‐1β activity, depending on cell type [108]. In our dataset, all participants maintained an IL‐1RN to IL‐1β ratio above 10 at every time point. However, while 50% and 56% of participants exceeded the >100 threshold at enrollment and postdelivery, respectively, this proportion dropped to 28% postpartum (Figure S12). Elevated IL‐1RN concentrations have been reported in certain pathological pregnancy‐related complications [90, 109, 110], though its anti‐inflammatory role suggests that this is likely a compensatory response rather than a direct cause of problems. In contrast, IL‐18 is potently proinflammatory, and is known to be more highly expressed in individuals living with HIV [111]. However, since ART has been shown to reduce IL‐18 concentrations [112], and the majority of our cohort was virally suppressed with postpartum IL‐18 levels showing a decline, HIV alone is unlikely to account for the elevated IL‐18 concentrations observed in our study. For more detailed reviews on immune factors in individuals living with HIV please refer to the following reviews: [26, 27, 28, 29, 30, 113].

The effects of ART on pregnancy outcome have been extensively studied, with some regimens linked to higher rates of adverse outcomes [114, 115, 116]. Although the ART combinations used in our study are considered safe for pregnant individuals [117], we tested whether the ART combination affected immune factor concentrations. Postdelivery and postpartum, individuals receiving the DTG‐based regimen had significantly higher concentrations of several inflammatory immune factors—including IFNγ, IL‐2, IFN‐α‐2, and IL‐18—compared to those receiving the EFV‐based regimen. However, consistent with previous findings, most immune factors were not significantly different by treatment group [118]. Previous research has demonstrated that various ART combinations, including DTG‐based regimens, are associated with distinct cytokine profiles [28, 52, 119]. Suggesting that one or both regimens in our study may influence inflammatory responses in the postpartum period. While we did not have weight data for participants, multiple studies have demonstrated greater weight gain over time with DTG‐based regimens compared to EFV‐based regimens [120, 121, 122], and elevated body weight is independently associated with increased inflammation [123, 124]. Despite this, pregnancy levels for the tested immune factors did not differ significantly between treatment groups, suggesting that there might be other factors that may contribute to the observed differences in immune factors postpartum. Notably, participants on EFV‐based regimens had longer median duration on ART (5 years) compared to those on DTG‐based regimens (3.1 years). Persistent inflammation can occur in individuals living with HIV, even when viral loads are undetectable [27, 28, 29, 30], though soluble immune profiles can change with continued treatment [125, 126]. Additionally, participants on a DTG‐based regimen were a median of 5 years older (33 years, IQR: 29–37) than those taking EFV (28 years, IQR: 27–35). Finally, a higher percentage of participants on the DTG‐based regimen had undetectable viral loads postdelivery (84%, *n* = 72) compared to those on EFV‐based regimen (60% *n* = 18). Taken together, these observations suggest that the differences in immune factor concentrations between treatment groups likely reflect a complex interplay of factors beyond ART regimen alone.

To properly contextualize these findings, it is important to acknowledge the limitations of this observational, pilot study. One key limitation is that this study was conducted within a larger cohort of pregnant individuals living with HIV, without a comparator group of individuals without HIV. We also have limited maternal health data to correlate immunologic parameters, such as CD4 T‐cell counts, blood pressure, and blood sugar. Where possible, we used historical immune factor data from other studies on people living with and without HIV to identify inconsistencies and outliers. Unfortunately, there are variations in concentrations of immune factors from study to study. This is a consequence of a variety of factors including: reported values (e.g., mean vs. median), method of detection [127], sample type (plasma vs. serum) [128, 129], anticoagulant used [129, 130], data handling, sample handling, and processing [42, 129, 131]. Additional factors such as timing of sample collection during the peripartum or postpartum [42, 45, 46, 132], pregnancy complications [37, 39, 133, 134, 135], the gravidity and parity of the participants [44, 87, 136] (Figure S13), and any of a variety of confounding factors external to and within the individual that can further complicate direct comparisons. Despite the variety of ways that our values can be skewed, most immune factors in our study were consistent with published literature [34, 50, 51, 72, 90, 91, 95, 101, 102, 137, 138, 139, 140, 141].

These challenges in standardizing immune factor measurements are compounded by the fact that the timing of delivery sample collection is often either unreported or variable across studies, making accurate comparisons difficult. Samples labeled as “delivery” can be collected anywhere from hospital admission to up to 2 weeks postpartum [72, 73, 74, 87, 94, 137, 138, 142, 143, 144]. In some studies, the timing differs between cohorts, with the group of interest sampled at one time point and the control group at another [138]. This variability between and within studies complicates data interpretation and synthesis. It presents a particular challenge, as research has shown that immune cell counts shift during labor [145] and that particular cell types change between 1 to 3 days postdelivery [146].

More recent studies further highlight the importance of timing in blood sampling around labor and delivery for immune factor analysis [42, 43, 44, 45, 147]. IL‐6, one of the most reported immune markers, increases from pre‐labor through labor [42, 45] then begins to decline by Day 1 postdelivery [45] continuing to decrease through at least Day 3 [43, 45]. In our study, we observed a significant increase in IL‐6 from enrollment to postdelivery. Notably, the median IL‐6 levels observed in our samples postdelivery were more comparable to the IL‐6 levels reported by Hebisch et al. at delivery rather than postdelivery. However, individuals with higher IL‐6 postdelivery were more likely to have a urinary tract infection or have a non‐elective C‐section [45]. Given that all participants in our study are living with HIV, this may partially explain the observed differences. Other studies examining IL‐6 levels in pregnancies affected by HIV have collected blood at the onset of labor [138]. Since the postdelivery sample timing is not consistent with what is most reported in the literature and the exact timing was not recorded, we are unable to directly correlate the immune factor concentration with sample timing or determine how each might change with time postdelivery.

Our findings, along with those from a few other studies, suggest that the immediate postdelivery period is a critical window for further investigation [43, 45, 146]. Pregnancy is often described as if it ends at labor and delivery, but the maternal body still undergoes dramatic physiological changes in the days and weeks after birth [71, 148, 149, 150]. There is very little known about the immune factors in the window from 1 to 3 days postdelivery, despite the importance of this window for postpartum hemorrhage, where risk factors are still widely unknown [151, 152]. Additionally, immune dysfunction has been linked to postpartum mental illnesses and hormonal imbalances [43, 153, 154, 155], and the postdelivery and postpartum periods are characterized by dramatic shifts in soluble immunity. A deeper understanding of these immune changes could offer valuable insights into postpartum disorders and help identify biomarkers for early detection and intervention.

## Ethics Statement

The authors confirm that the ethical policies of the journal, as noted on the journal's author guidelines page, have been adhered to and the appropriate ethical review committee approval has been received. The study conformed to the US and DRC Policies for the Protection of Human Subjects. This is an observational cohort study within a larger study approved by Institutional Review Boards (IRB) at The Ohio State University (IRB study ID: 2015H0440), Albert Einstein College of Medicine (protocol #2020‐12018), and the Kinshasa School of Public Health Ethics Committee (protocol #0001 1–04101‐00001365292–20). Written informed consent was obtained directly from participants. Data were de‐identified prior to processing and analysis.

## Conflicts of Interest

J.C., N.Z., M.T., C.K.C., F.L.K., B.L.M., P.B., N.P.M.L., M.Y., and J.J.K. declare no conflicts of interest. N.T.F. has received funding from Gilead unrelated to this study.

## Supporting information




**Supplementary File 1:** aji70147‐sup‐0001‐SuppMat.docx.

**Supplementary File 2:** aji70147‐sup‐0002‐TableS1‐S6.xlsx.
