# Supplementary material for: Dynamics of Cytokines and Chemokines During the Peripartum Period in People Living With Human Immunodeficiency Virus
Source: Am J Reprod Immunol. 2025 Aug 22;94(2):e70147. doi: 10.1111/aji.70147 (PMC12372876; doi:10.1111/aji.70147)
Supplement: Supplementary file 1 — aji70147‐sup‐0001‐SuppMat.docx. [file AJI-94-e70147-s002.docx]

**Supplementary Material**

Table S1. Immune factor nomenclature and assay info.

Table S2. Assay limits for each immune factor for each time point. Abbreviations: L-LoD-Lower-Limit of detection; DF-Dilution factor; U-LoD-Upper-Limit of detection. In the columns % below (LoD) and % above (LoD) the values highlighted red are >75%, the values highlighted yellow are between 50 and 75%. Samples with >50% of samples outside of detection limit for all samples were not included in main figures.

Table S3. Descriptive statistics.

Table S4. Values associated with Figure S9. Enrollment Spearman correlation p-values and Spearman r-values. For p-values the yellow color represents a p>0.05, the green represents values p>0.01. For Spearman’s r-values, green represents values r>0.5, red represents r<-0.5. The number of values in each row are tabulated to the right, values >0.5 and values <-0.5. The total number of values >0.4, >0.7, >0.8 and <-0.4 are tabulated at the bottom.

Table S5. Values associated with Figure S10. Postdelivery Spearman correlation p-values and Spearman r-values. For p-values the yellow color represents a p>0.05, the green represents values p>0.01. For Spearman’s r-values, green represents values r>0.5, red represents r<-0.5. The number of values in each row are tabulated to the right, values >0.5 and values <-0.5. The total number of values >0.4, >0.7, >0.8 and <-0.4 are tabulated at the bottom.

Table S6. Values associated with Figure S11. Postpartum Spearman correlation p-values and Spearman r-values. For p-values the yellow color represents a p>0.05, the green represents values p>0.01. For Spearman’s r-values, green represents values r>0.5, red represents r<-0.5. The number of values in each row are tabulated to the right, values >0.5 and values <-0.5. The total number of values >0.4, >0.7, >0.8 and <-0.4 are tabulated at the bottom.


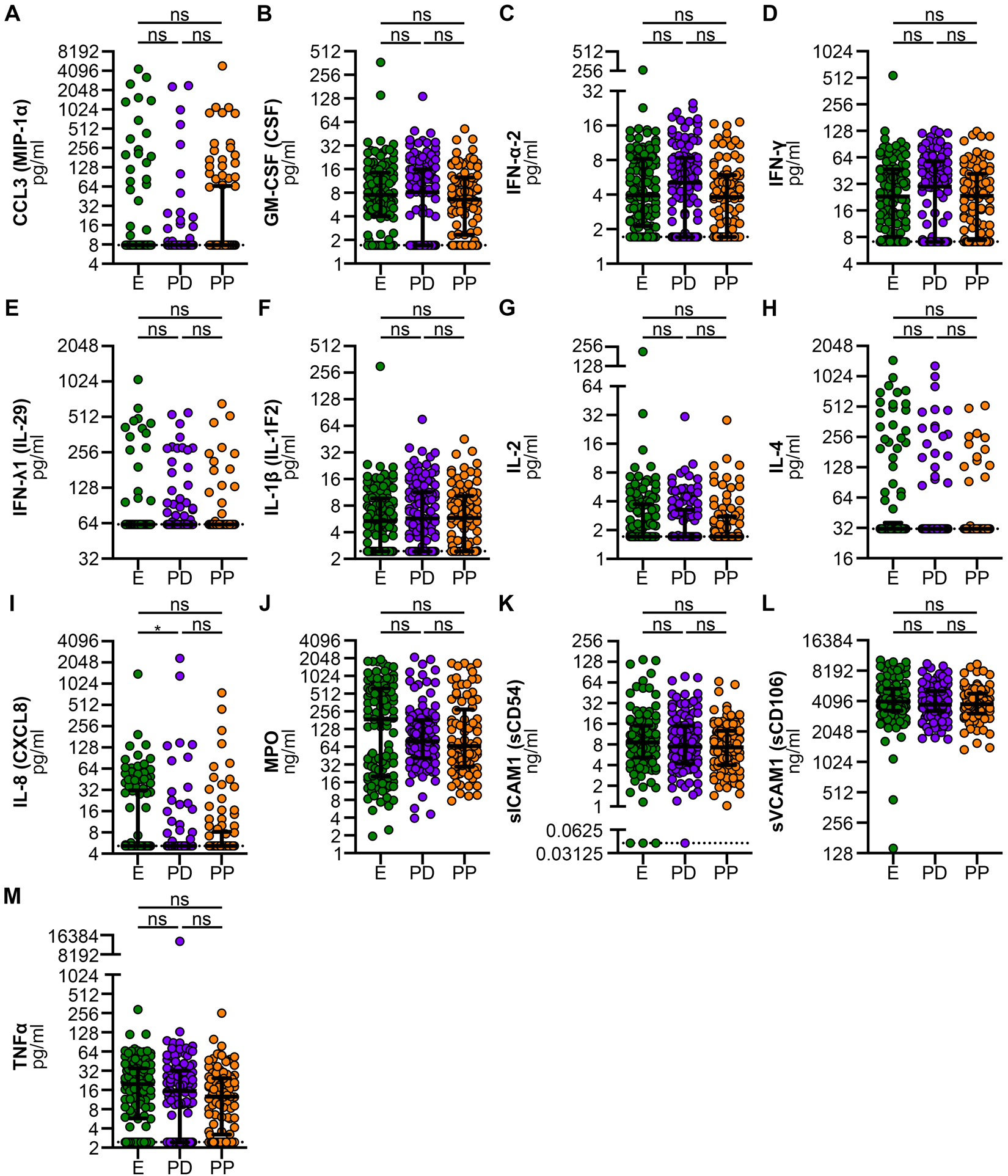


Figure S1. Immune factors that do not change significantly across pregnancy. Immune factors were measured in plasma collected from participants at enrollment (E), postdelivery (PD), and postpartum (PP) using LEGENDplex assays (A-D, F-G, I-M) or ELISA (E, H). Immune factor concentrations plotted as pg/ml (A-I, M) or ng/ml (J-L). (A-M) Each dot represents an individual participant’s analyte measurement with horizontal lines indicating the median, upper and lower quartiles. Dotted lines represent LoD, see Supplemental Table 2 for all LoD values. Statistical significance tested by Kruskal-Wallis with a Dunn’s correction for multiple comparison. ns=not significant, *p<0.05.


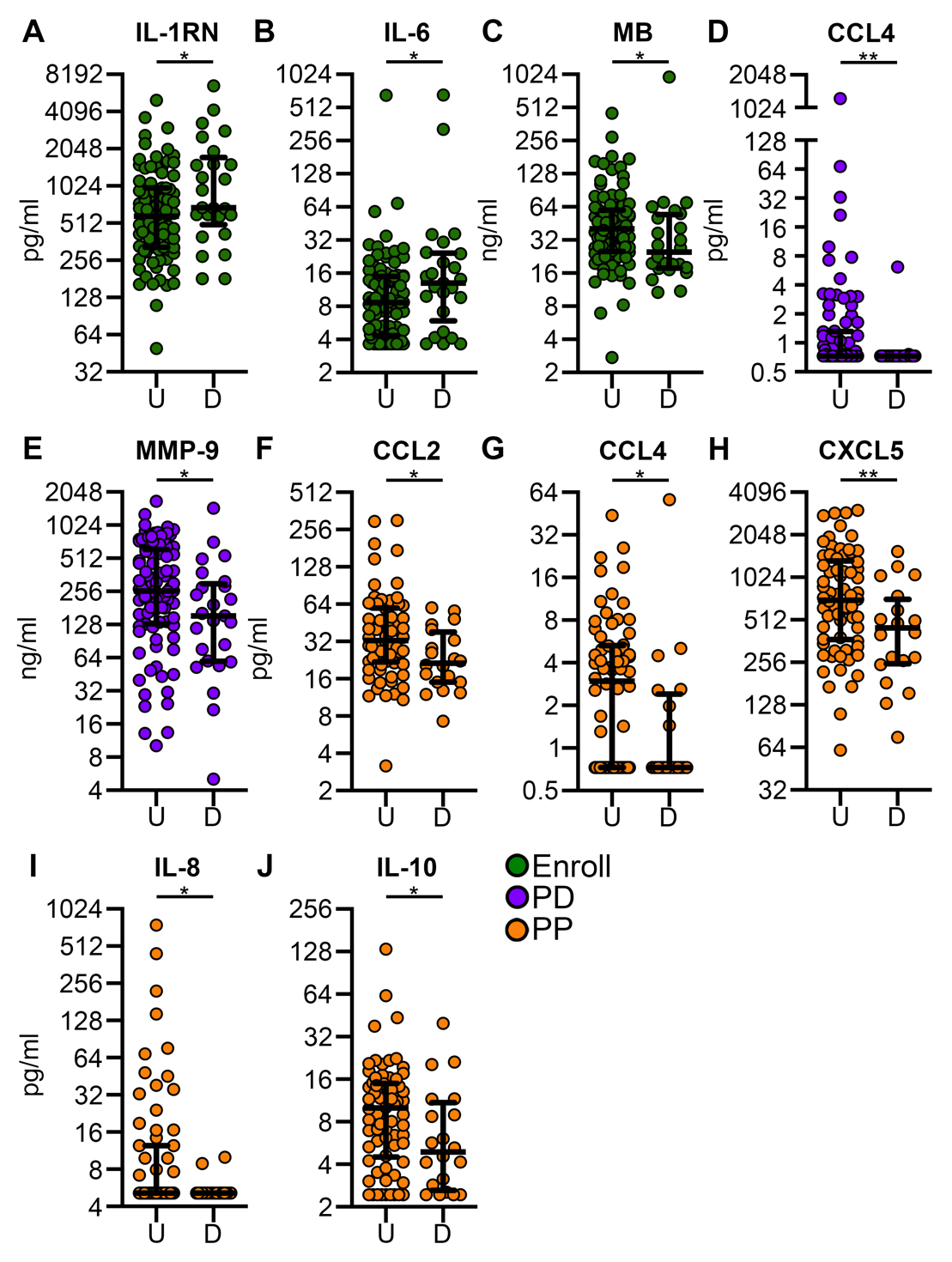


Figure S2. Immune factors vary significantly with HIV detection. Immune factors measured as before at (A-C) enrollment (Enroll, green), (D-E) postdelivery (PD, purple) or (F-J) postpartum (PP, orange) were compared between those individuals that had undetectable (U) HIV copies (<40 copies/ml) or detectable (D) HIV copies (≥40copies/ml). Each dot represents an individual participant’s analyte measurement, horizontal lines represent the median, upper and lower quartile. Significance was measured via Mann-Whitney test, *p<0.05, **p<0.01.


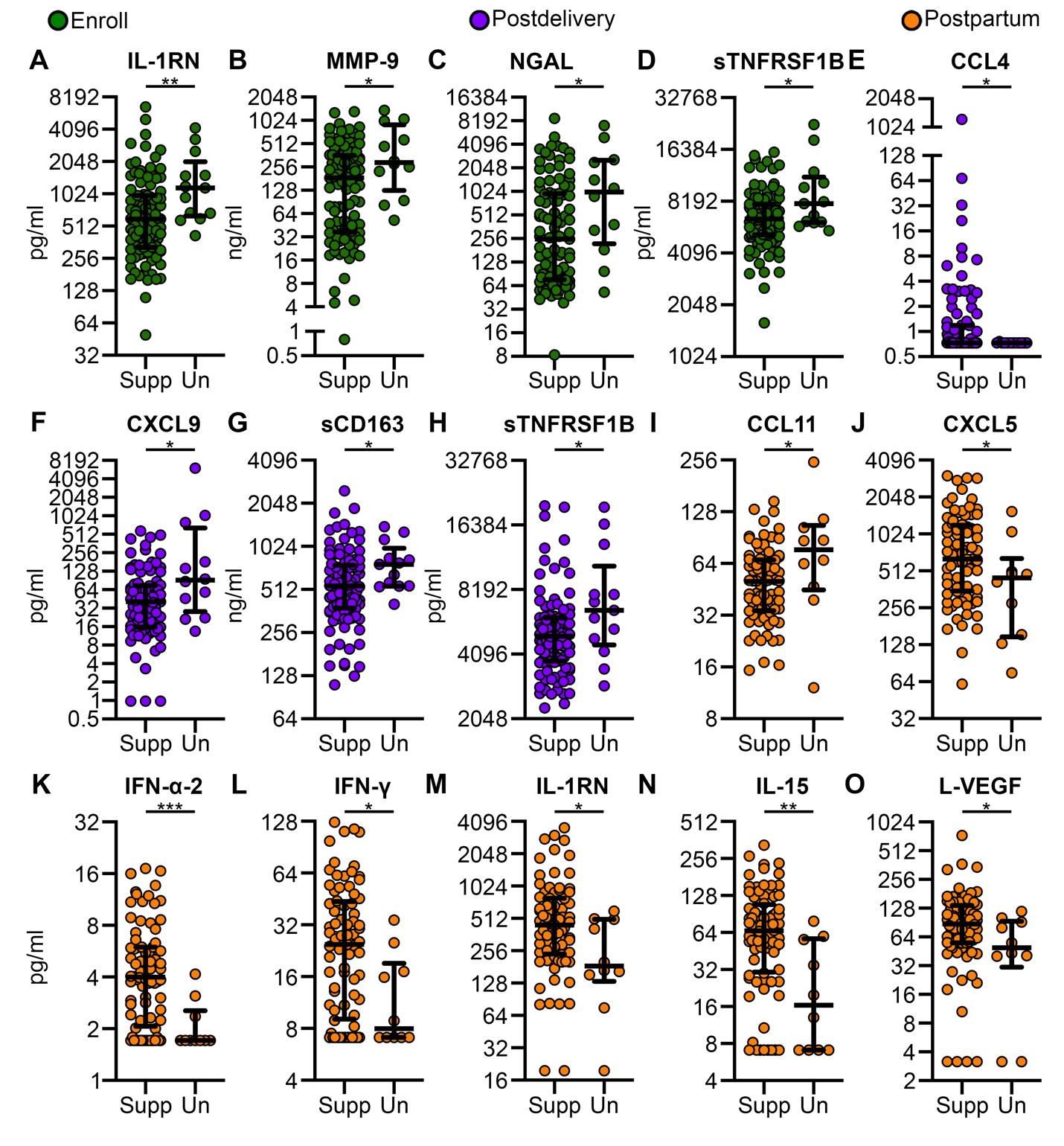


Figure S3. Immune factors that vary significantly with HIV suppression. Immune factors measured as before at (A-D) enrollment (green), (E-H) postdelivery (purple) or (I-O) postpartum (orange) were compared between those individuals that were suppressed (Supp) (<1000 HIV copies/ml) or unsuppressed (Un) (≥ 1000 HIV copies/ml). Each dot represents an individual participant’s analyte measurement, horizontal lines represent the median, upper and lower quartile. Significance was measured via Mann-Whitney test, *p<0.05, **p<0.01, ***p<0.001.


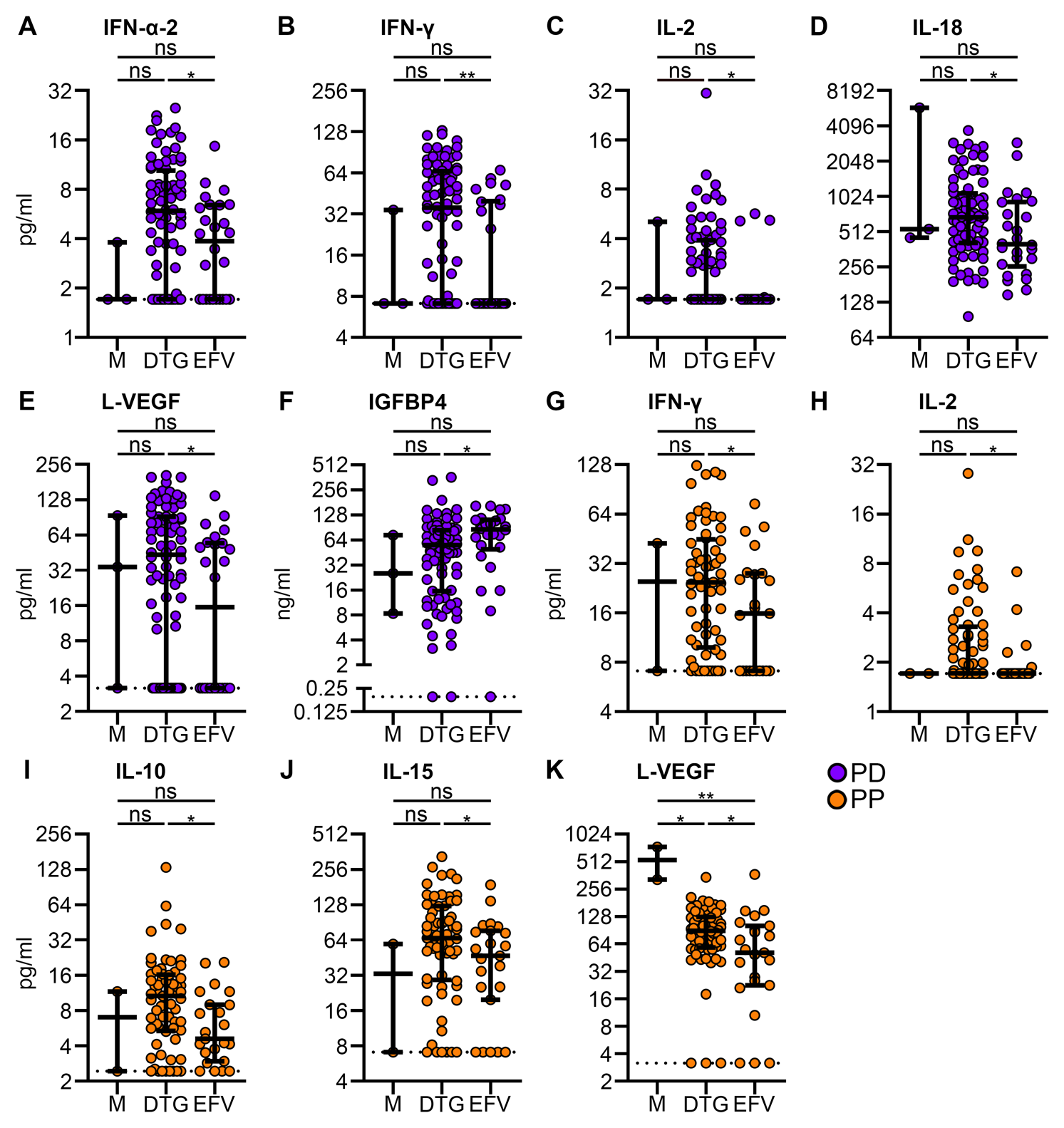


Figure S4. Cytokines and soluble factors that are significantly different by ART regimen. Cytokines and soluble factors measured as before at (A-F) postdelivery (PD, purple) or (G-K) postpartum (PP, orange) were compared between those individuals whose ART-regimen was missing (M), with those on a dolutegravir (DTG)-based regimen and those on an efavirenz (EFV)-based regimen. Each dot represents an individual participant’s analyte measurement, horizontal lines represent the median, upper and lower quartile. Dotted lines represent LoD. Significance was measured via Mann-Whitney test with no adjustment for multiple comparisons. ns=not significant, *p<0.05, **p<0.01.


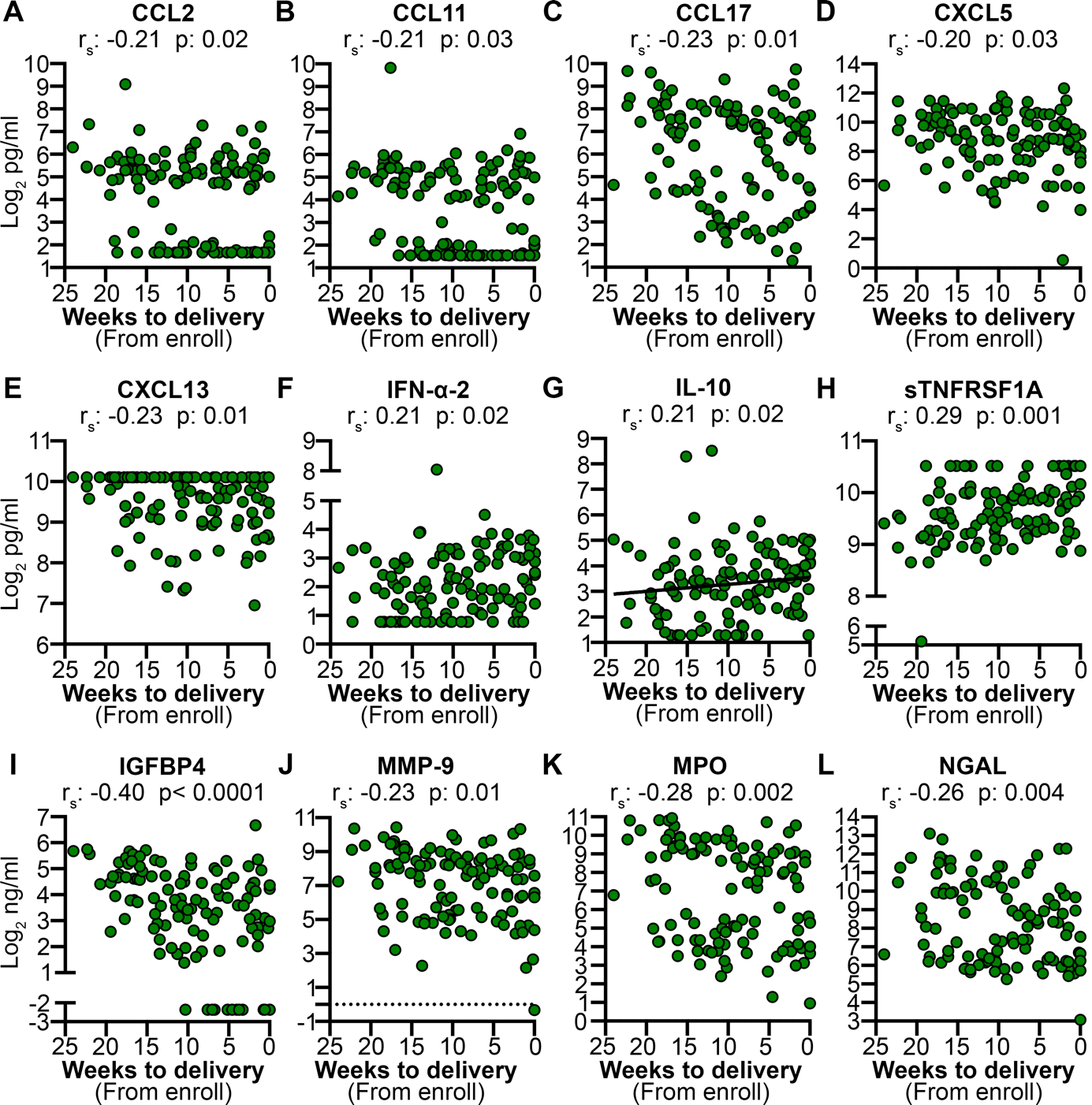


Figure S5. Concentration of immune factors measured at enrollment that significantly associate with time to delivery. Immune factors were measured as described at enrollment. (A-K) Log_2_ concentration of immune factors at enrollment were plotted against the number of weeks between enrollment and delivery. Each dot represents an individual participant’s analyte measurement. Statistical significance tested by Spearman correlation (r_S_) and approximate p-value of the correlation displayed. Only those with a p-value<0.05 and an r_S_>0.2 or <-0.2 were considered significant.


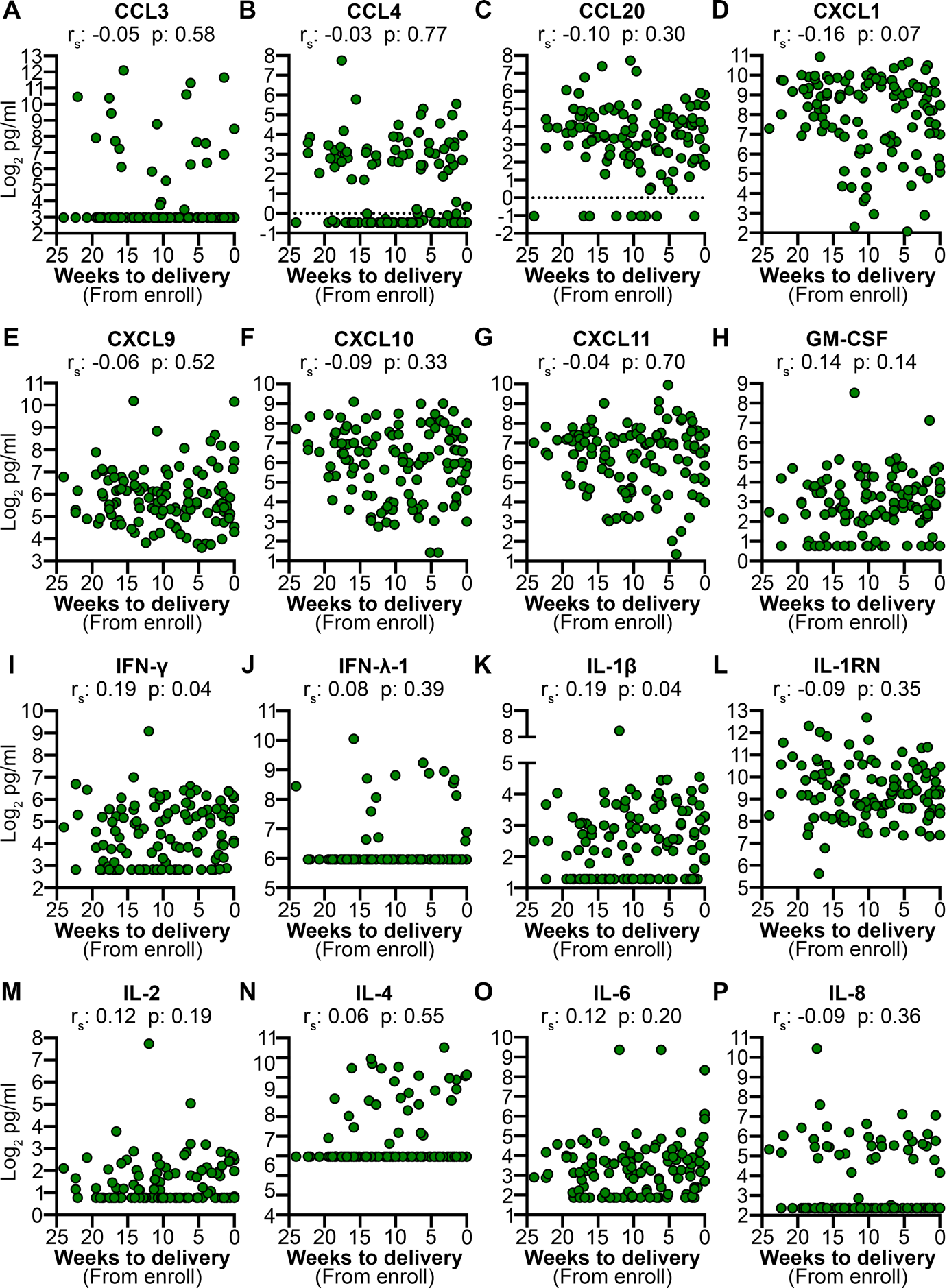


Figure S6. Concentration of cytokines and chemokines measured at enrollment that are unassociated with time to delivery. Cytokines and chemokines were measured as described at enrollment. (A-P) Log_2_ concentration of chemokine or cytokine at enrollment were plotted against the number of weeks between enrollment and delivery. Each dot represents an individual participant’s analyte measurement. Statistical significance tested by Spearman correlation (r_S_) and approximate p-value of the correlation displayed.


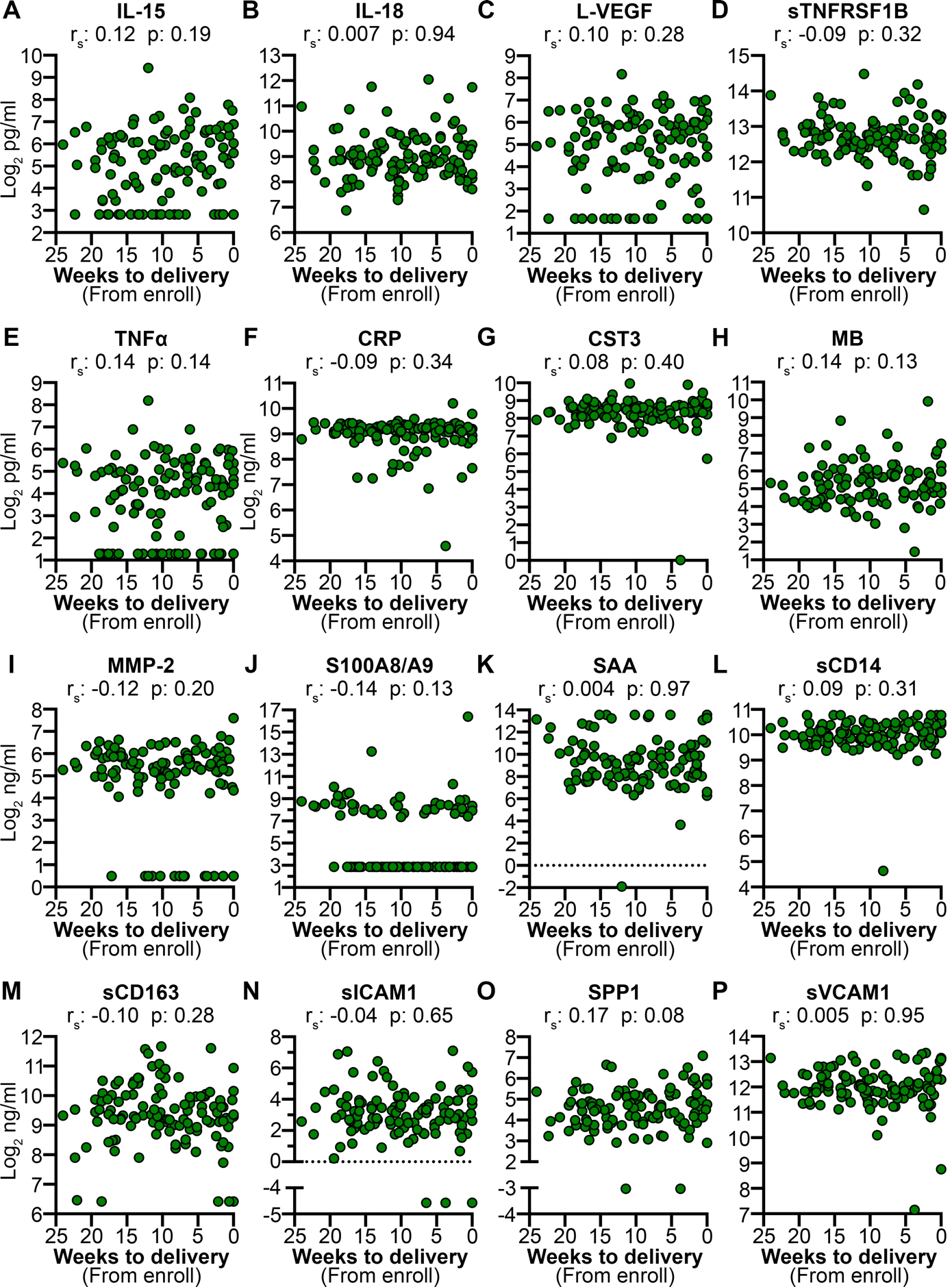


Figure S7. Concentration of immune factors measured at enrollment that are unassociated with time to delivery. Immune factors were measured as described at enrollment. (A-P) Log_2_ concentration of chemokine, cytokine or soluble factor at enrollment were plotted against the number of weeks between enrollment and delivery. Each dot represents an individual participant’s analyte measurement. Statistical significance tested by Spearman correlation (r_S_) and approximate p-value of the correlation displayed.


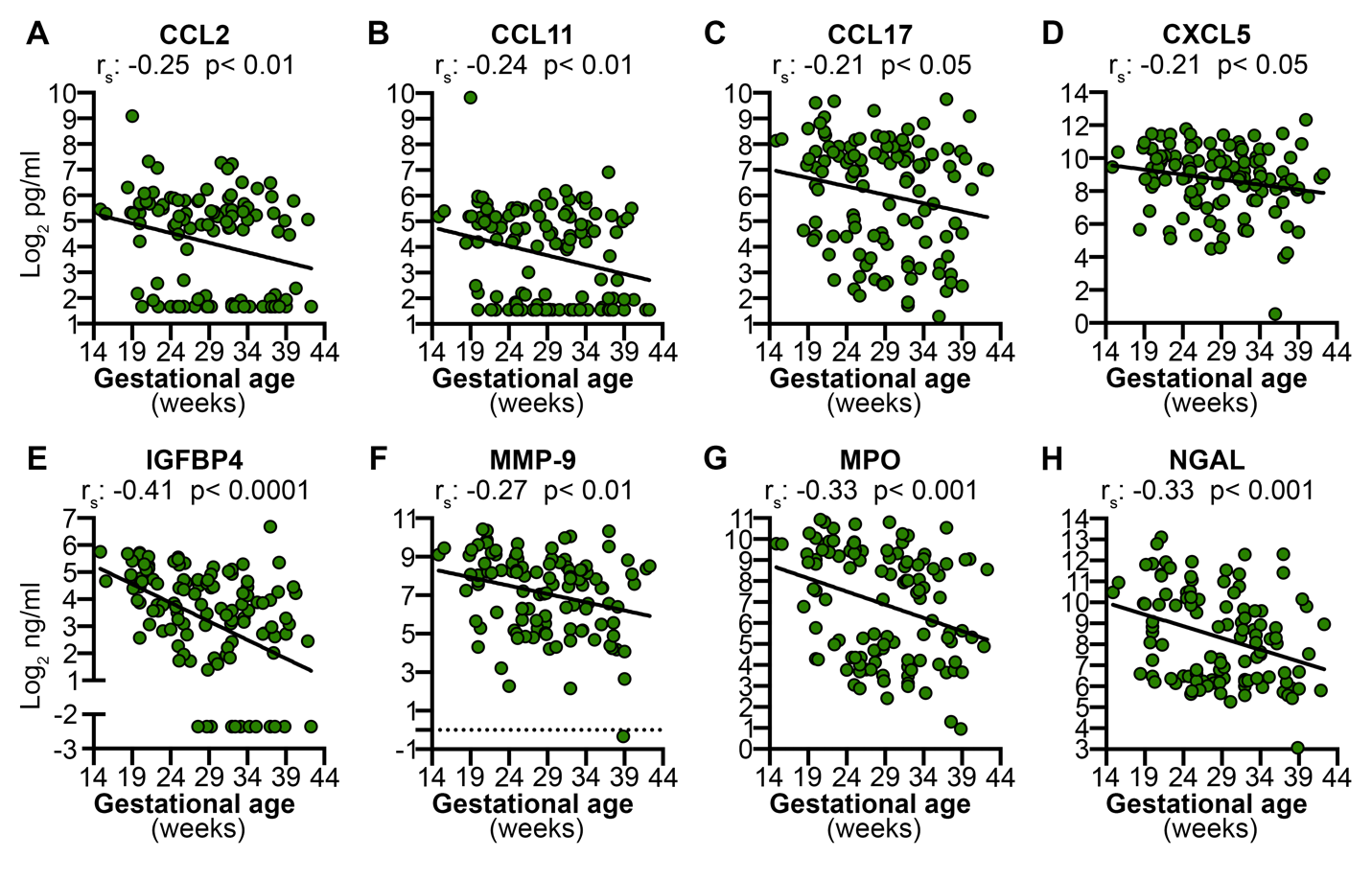


Figure S8. Concentration of immune factors measured at enrollment that significantly associate with gestational age. Immune factors were measured as described at enrollment. (A-K) Log_2_ concentration of immune factors at enrollment were plotted against the gestational age at enrollment. Each dot represents an individual participant’s analyte measurement. Line generated using simple linear regression. Statistical significance tested by Spearman correlation (r_S_) and approximate p-value of the correlation displayed. Only those with a p-value<0.05 and an r_S_>0.2 or <-0.2 were considered significant.


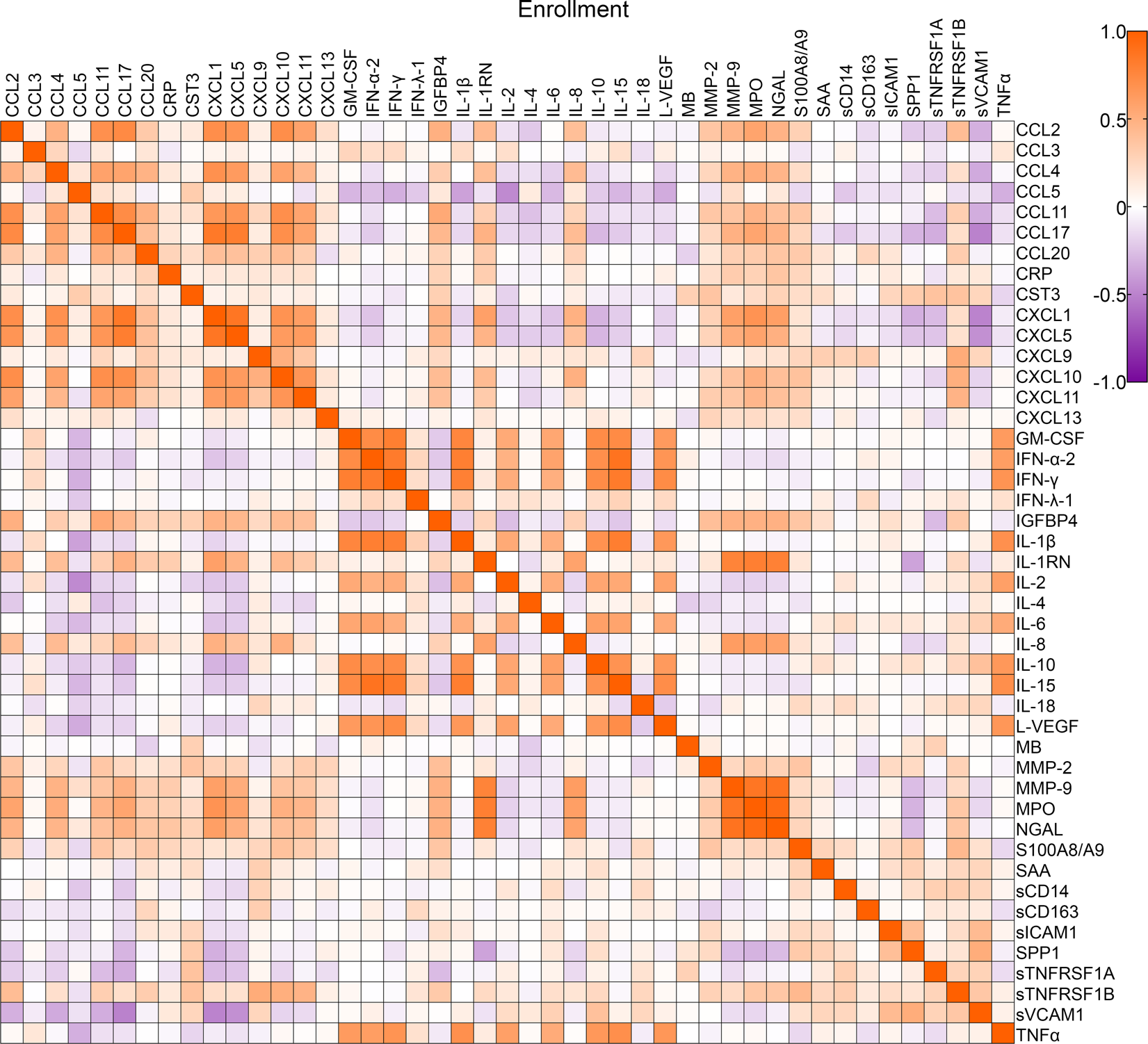


Figure S9. Correlations at enrollment. Graphical representation of Spearman correlations between immune factors in participants’ plasma at enrollment. Spearman r and p-values are in Supplemental Table 4.


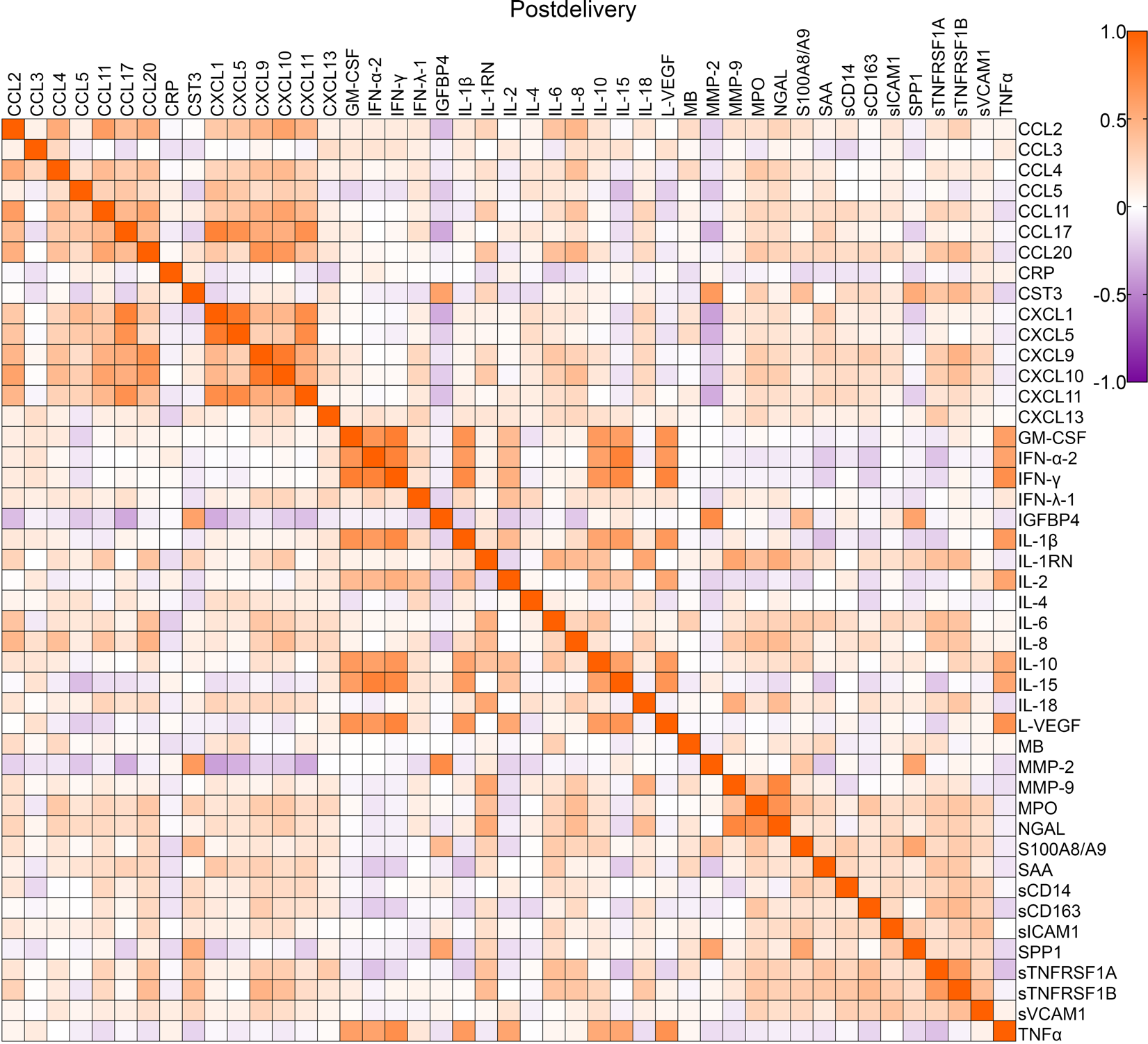


Figure S10. Correlations at 1-3 days postdelivery. Graphical representation of Spearman correlations between immune factors in participants’ plasma 1-3 days postdelivery. Spearman r and p-values are in Supplemental Table 5.


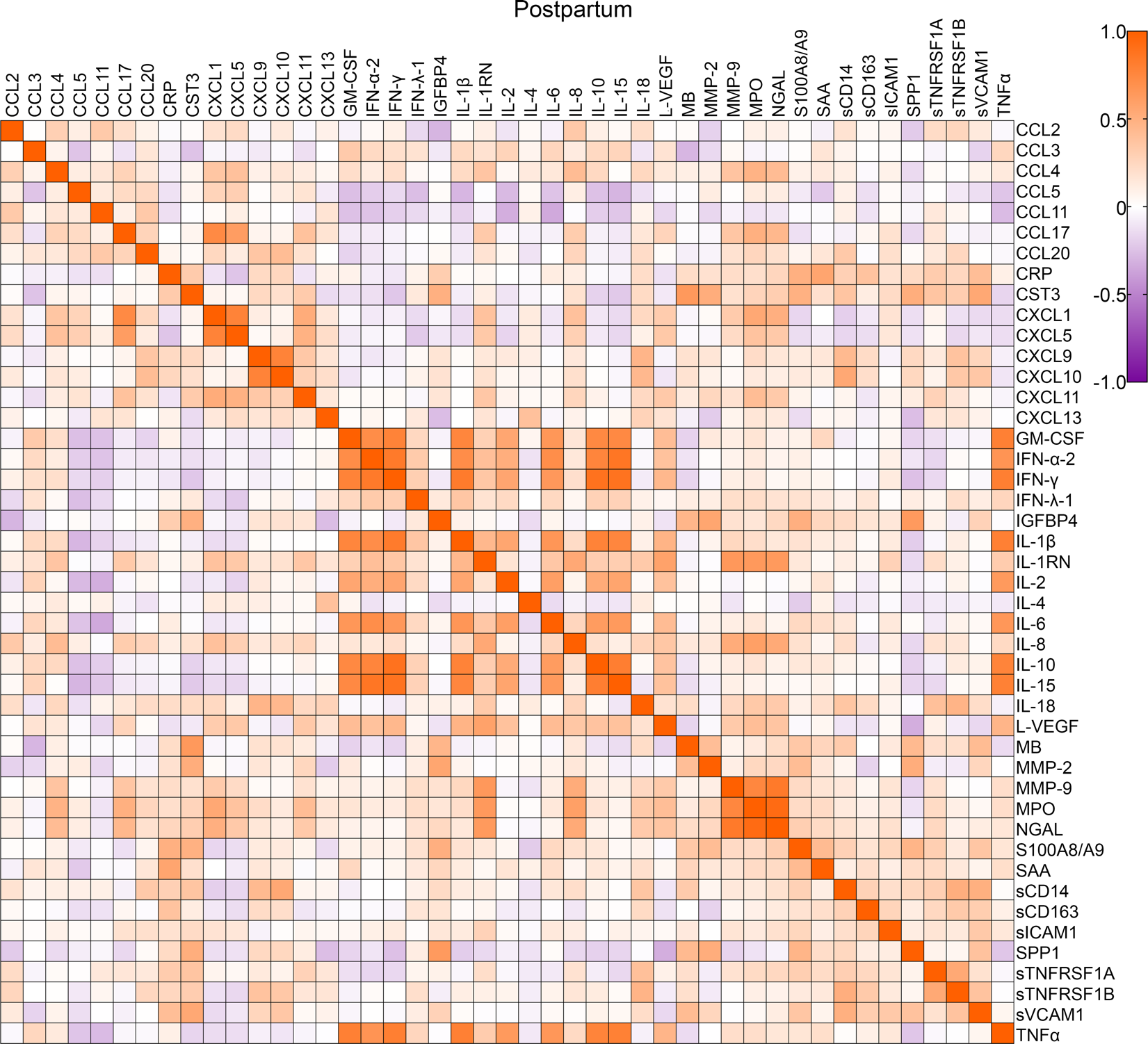


Figure S11. Correlation at median 6.7 weeks postpartum. Graphical representation of Spearman correlations between immune factors in participants’ plasma postpartum. Spearman r and p-values are in Supplemental Table 6.


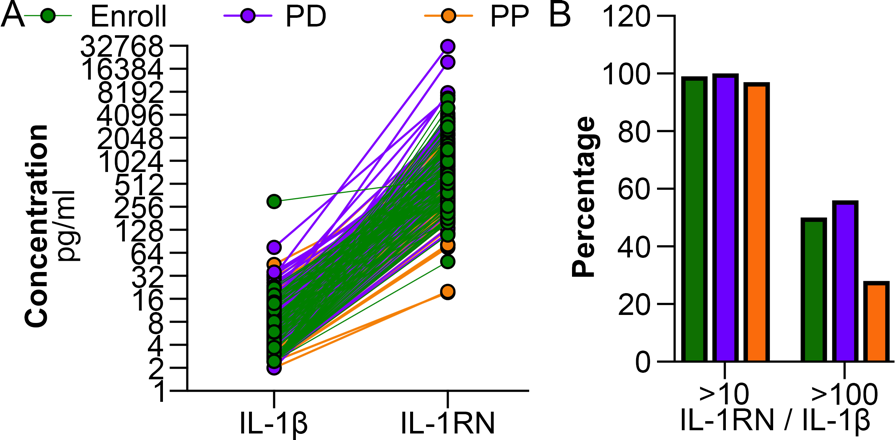


Figure S12. IL-1RN levels are higher than IL-1β levels. (A) Graph displaying the concentrations of IL-1β and IL-1RN at enrollment (enroll, green), postdelivery (PD, purple) and postpartum (PP, orange). Each dot represents an individual participant’s analyte measurement. (B) Percentages of samples that have a ratio of IL-1RN/IL-1β >10 and >100.


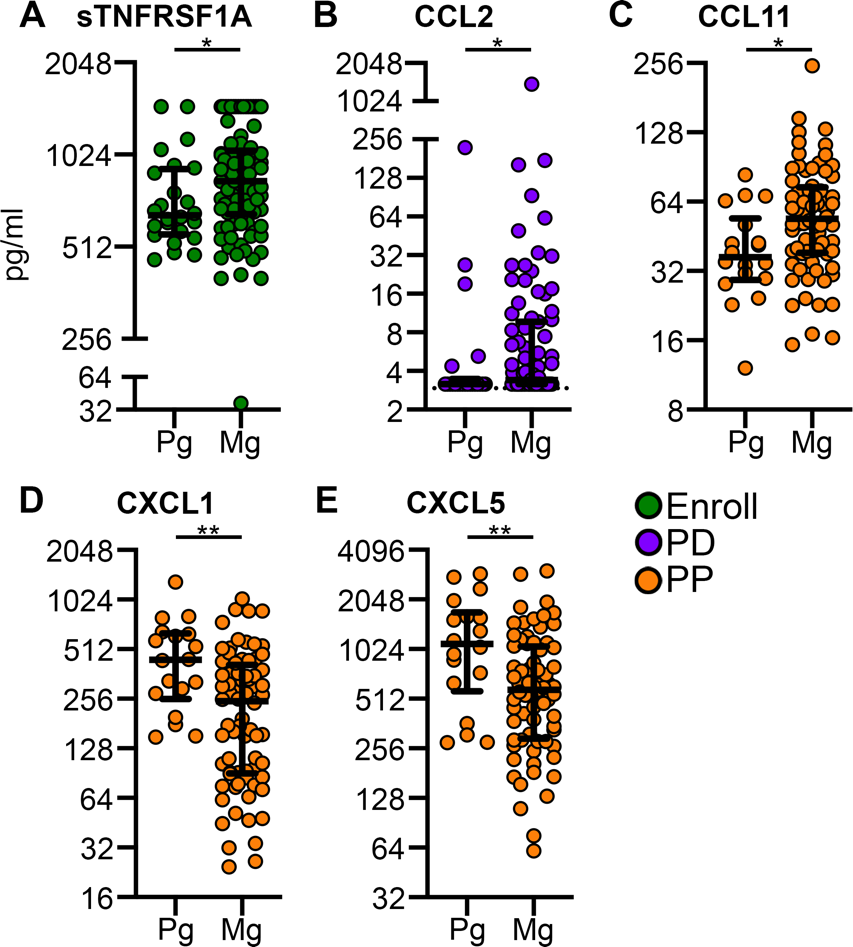


Figure S13. Immune factors that are significantly different by gravidity. Immune factors measured as before at (A) enrollment (green), (B) postdelivery (PD, purple) or (C-E) postpartum (PP, orange) were compared between primigravid (Pg) participants and multigravid (Mg) participants. Each dot represents an individual participant’s analyte measurement, horizontal lines represent the median, upper and lower quartile. Dotted lines represent LoD. Significance was measured via Mann-Whitney test, *p<0.05, **p<0.01.
